# The Role of *Guanxi* and Positive Emotions in Predicting Users’ Likelihood to Click the Like Button on WeChat

**DOI:** 10.3389/fpsyg.2020.01736

**Published:** 2020-07-22

**Authors:** Haichuan Zhao, Mingyue Zhang

**Affiliations:** ^1^School of Management, Shandong University, Jinan, China; ^2^Asia Europe Business School, Faculty of Economics and Management, East China Normal University, Shanghai, China

**Keywords:** intention to like, *guanxi*, affective cues, positive emotion, expected *guanxi* benefit

## Abstract

Clicking the like button is a popularly used interaction function on social media. Although prior empirical studies have examined motives for liking behavior on social media, they have largely neglected culture-driven theories and the underlying mechanisms for why users click the like button. By integrating *guanxi* theory and the affective response model, we propose a conceptual model to determine what factors influence users’ liking behavior on WeChat. We examine data from an online survey of 327 respondents using PLS-SEM. The findings show that content cues (i.e., content usefulness and content interestingness) positively affect WeChat users’ positive emotions, which in turn predict their intention to like. More importantly, *guanxi* cues (i.e., *mianzi* giving, *renqing*, and *ganqing*) positively affect WeChat users’ expected *guanxi* benefit, which in turn affects intention to like in two ways. On the one hand, the expected *guanxi* benefit has a direct positive effect on intention to like; on the other hand, this benefit can translate into positive emotions, which in turn predict users’ intention to like.

## Introduction

The advancement of the Internet has increased the popularity of social media. In turn, social media has significantly changed interpersonal interactions. The like button is a popularly used interaction function on social media (Basalingappa et al., 2016), such as Facebook, Twitter, Sina micro-blog, and WeChat, and other online platforms also provide this function. The like button was initially supposed to show users’ positive emotions about the usefulness and interestingness of the content (Sun et al., 2016); however, with the increased usage of this button, the original meaning of “like” has changed. Many users now assume that the number of likes received from others is a reflection of reputation and social status. Thus, they also use the like button to maintain and strengthen relationships with others by showing support (Wang et al., 2016). By exploring the complicated motivations behind the use of the like button, this research tries to uncover the factors and mechanisms that influence people’s use of this button on social media from a *guanxi* and emotional perspective.

Users’ liking behavior on social media has received some attention in the literature under two main research streams. The first pertains to the consequences of users’ liking behavior from a company marketing perspective. Prior research indicates that customers’ use of the like button can increase their word-of-mouth and purchase intentions (Bunker, 2013; Mariani and Mohammed, 2014). The second stream involves users’ motivations for clicking the like button from an interpersonal interaction perspective (Lee et al., 2016; Sun et al., 2016; Gan, 2017). For example, Sun et al. (2016) find that social value, entertainment value, and cognitive value are key determinants of WeChat users’ liking behavior. Gan (2017) argues that user-perceived hedonic gratification, social gratification, and utilitarian gratification positively affect WeChat users’ liking behavior. WeChat is the most popular form of social media in China. It provides multiple mobile services, including mobile communication services via text and voice and mobile social networking services. Although prior research has examined users’ liking behavior in a Chinese context, it has neglected some key elements that may affect Chinese users’ behavior, such as *guanxi*. The Chinese use WeChat not only to express their emotions when reading useful and interesting content but also to strengthen *guanxi* with their friends (Wang et al., 2016). *Guanxi*, or the existence of direct particularist ties between two or more individuals (Jacobs, 1979; King, 1991; Tsui and Farh, 1997), plays a major role in Chinese society (Alston, 1989) and is considered an important strategic asset by most Chinese people.

Although a few previous studies examined the impact of such social dimensions as social gratification and social value on WeChat users’ liking behavior (Sun et al., 2016; Gan, 2017), *guanxi* is a conceptual construct that can be clearly distinguished from social gratification and social value. Social value refers to social benefits related to social interaction such as reciprocity, altruism, and expected relationship (Sun et al., 2016), whereas social gratification emphasizes maintaining social relationships by fulfilling social support by liking behavior in WeChat (Gan, 2017). In comparison, *guanxi* encompass three dimensions such as *renqing* (reciprocity), *mianzi* (face) giving, and *ganqing* (affect). *Mianzi* (face) giving refers to social image maintenance and construction by liking each other’s post in WeChat and *ganqing* (affect) indicate that people use liking behaviors in WeChat to confirm the closeness of their relationships. Thus, *guanxi* goes beyond the conceptual scope of social value and social gratification, and furthered the field of liking behavior in WeChat by approaching it in the perspective of social image and affective concerns. Thus, we examine how *guanxi* affects users’ liking behavior on social media. In addition, prior research only specifies the factors that affect users’ likelihood to click the like button, while neglecting the underlying mechanisms that may affect users’ liking behavior. This study aims to fill these gaps.

When people decide to click the like button, they are considering more than just how the content makes them feel. Their decision is also likely based on both cognitive factors (e.g., content quality, expected *guanxi* benefit) and affective factors (e.g., how it makes them feel, or positive emotions). Thus, building on the affective response model (ARM) and *guanxi* theory, this paper proposes an integrated conceptual model that shows how *guanxi* cues (i.e., *mianzi* giving, *renqing*, and *ganqing*), content cues (content usefulness and content interestingness), and expected *guanxi* benefit motivate users to click the like button due to positive emotions. We empirically test the model and hypotheses using data from a WeChat sample.

## Theoretical Background and Research Hypotheses

### Linguistic and Individual’s Behavior

WeChat users often post information on WeChat Moments. Language, in a broad sense, is the way information is expressed. Users’ language used in a post may express their idea, mood, and experience, and it may affect their friends’ reactions to the post, such as clicking the “like” button, writing reviews, and re-post. Previous research indicated that the language will influence reader’s social cognition and emotion, and further affect their behavior (Burke, 1966; Semin and Fiedler, 1988; Leiner et al., 1991; Pennebaker and Francis, 1996; Mascolo and Fischer, 2015). Furthermore, prior research suggested that people’s cognition on language was also affected by their culture which was developed by historical circumstances (Gobel et al., 2011; Tajima and Duffield, 2012). In Chinese society *guanxi* is considered an important factor that shape Chinese’s interaction with others, and their cognition, emotion, and behavior can be affected by *guanxi.* Thus, in our paper we chose guanxi cues (culture related cues) and content cues (linguistic related cues) as two kinds of antecedents of the behavior of clicking the “like” button.

### Affective Response Model (ARM)

Affect is a critical factor in human decisions and behaviors in many social contexts. Zhang (2013) developed ARM to explain how people’s affect influences their decisions and behavior within the information and communication technology (ICT) context. WeChat, a popular ICT in China, provides instant messages via text and voice and mobile social networking services (Che and Cao, 2014; Zhang et al., 2016; Gan, 2017; Gan and Li, 2018). Thus, we adopt ARM to explain users’ liking behaviors in this context. ARM proposes that ICT stimuli (e.g., affective cues) can influence people’s affective reactions (e.g., emotions). These affective reactions generate and arouse people’s behavior in the ICT. Research also indicates that people make final decisions and correspondingly choose their behavioral responses under different emotional states, such as approach versus avoidance (Mehrabian and Russell, 1974). In the context of liking behavior, the reason people click on the like button is to show their appreciation of others or others’ posts, and this positive emotion induces their liking behavior.

In the ARM, affective cues are “specific features or characteristics of a stimulus that can manifest the affective quality of the stimulus” (Valdez and Mehrabian, 1994; Zhang, 2013). Affective cues have been assessed as environmental cues or signals containing affective information that can influence emotions (Valdez and Mehrabian, 1994) and cognitive processing strategies (Soldat et al., 1997). The literature specifies that information characteristics (Wang et al., 2017), environmental characteristics (Valdez and Mehrabian, 1994), product characteristics (Graa and Danielkebir, 2012), and social relationship characteristics (Wang et al., 2017) are all distinctive affective cues. As mentioned previously, people use the like button not only to express their positive emotions about the usefulness and interestingness of the content but also to maintain and strengthen *guanxi* with others (Wang et al., 2016; Sun et al., 2016). Building on the literature on affective cues, we identify the affective cues that fit the context of WeChat and categorize them into two types: *guanxi* cues (i.e., *mianzi* giving, *renqing*, and *ganqing*) and content cues (i.e., content usefulness and content interestingness). We then explain our main reasons for choosing these characteristics.

### *Guanxi* Theory

According to previous research, *guanxi* is a social exchange mechanism built on mutual favors (Lee et al., 2017). The concept of guanxi is rooted in Confucianism; it is considered an important strategic asset by most Chinese people and companies (Park and Luo, 2010; Lee et al., 2017). *Guanxi* has been examined in both business and interpersonal relationships. Previous studies in businesses have mainly identified *guanxi* networks as a resource for organizations through which potential buyers and sellers are identified in China (Park and Luo, 2010; Yen et al., 2017). That research has mainly focused on the outcomes of *guanxi*, such as business performance, relationship-specific investment, and knowledge sharing (Zhuang and You-Min, 2007; Park and Luo, 2010; Luo et al., 2012; Yen et al., 2017). Previous research in interpersonal relationship has mainly explored how to build interpersonal *guanxi* (Ying, 2002; Warren et al., 2004; Chen et al., 2012). These studies indicate that Chinese people often participate in social activities to develop their *guanxi* networks with those who have the same friends, the same hometown, the same school, and the same neighborhood (Lee et al., 2001). In the process of developing *guanxi*, the Chinese tend to engage in the appropriate actions to enhance and maintain their *guanxi* networks, such as *mianzi* (face) giving, *renqing* (favor) giving, and *ganqing* (affect) supporting (Hwang, 1997); in return, they also expect to receive the *guanxi* benefit in return when they engage in actions to develop *guanxi*. WeChat is a social platform on which the Chinese can maintain and enhance *guanxi* with others in their social networks (Wang et al., 2016). Thus, we identify users’ *guanxi* motivation as an affective cue of positive emotions and intention to like.

### *Guanxi* Cues

#### *Mianzi* Giving and Expected Positive *Guanxi* Benefit

*Mianzi* (face) refers to an individual’s claimed sense of positive self-image of his or her social status (Lee and Dawes, 2005). In general, the Chinese are concerned about saving face themselves and also giving *mianzi* to others (Redding and Michael, 1983). Previous research indicates that *mianzi* can be shared (Haugh and Hinze, 2003). The Chinese often expect members of their social networks to give *mianzi* to one another (Cardon and Scott, 2003). That is, it is the responsibility of all members in the network to enhance one another’s status. Enhancing and maintaining *mianzi*, in turn, improves the *guanxi* between members (Cardon and Scott, 2003). On WeChat, many people consider that they have a great deal of *mianzi* when their posts receive many likes, and vice versa. The Chinese want to maintain or enhance the *mianzi* of others because providing no *mianzi* can destabilize or end the *guanxi* between members (Wang et al., 2016). Thus:

H1: *Mianzi* giving positively influences WeChat users’ expected *guanxi* benefit.

#### *Renqing* and Expected Positive *Guanxi* Benefit

*Renqing*, or reciprocity, refers to one’s obligation to return favors and show empathy to his or her personal relationship partner (Wang, 2007). More specifically, it means that if a personal relationship partner has received a favor, he or she must return the favor as soon as the opportunity arises (Hwang, 1987; Lovett et al., 1999). When both parties of a personal relationship engage frequently in the exchange of *renqing*, their *guanxi* will be strengthened. On WeChat, users can give their social network partner a favor by clicking the like button. They may also be recipients of others’ likes by engaging in liking behavior for others. The rules of reciprocity in liking behavior thus enhance users’ *guanxi* with others.

H2: *Renqing* positively influences WeChat users’ expected *guanxi* benefit.

#### *Ganqing* and Expected Positive *Guanxi* Benefit

*Ganqing* (affect), which is an indicator of the closeness of *guanxi*, refers to the feelings of emotional commitment among members of a social network (Mei-hui Yang, 1994). It is a form of social capital that provides leverage in interpersonal exchanges of favors (Yen et al., 2011). *Ganqing* can be built by sharing feelings with others about common topics, such as hobbies, experiences, beliefs, judgments, and opinions (Chen et al., 2004). On WeChat, if others share their experiences, opinions, and so on, in their posts, clicking the like button means that users endorse the content in those posts; thus, two parties can obtain *ganqing* on a long-term basis through liking behavior.

H3: *Ganqing* positively influences WeChat users’ expected *guanxi* benefit.

### Content Cues

Many users use WeChat to obtain useful and helpful content (Gan and Li, 2018). Previous research indicates that the content of a post is the key determinant of people’s choice of behavior toward that post, such as reading, writing a review, reposting, and clicking the like button (Zhao et al., 2016a, b). Gan (2017) shows that useful content seeking motivates users’ liking behavior; thus, content is an important cue that influences users’ liking behavior. Zhao et al. (2016a) proposes that users evaluate the content from two aspects: usefulness and interestingness. Accordingly, we chose these two aspects as affective cues that influence users’ emotions and liking behavior.

*Content usefulness* refers to the degree to which an online post provides useful content that gives economic or functional benefits to users (Zhao et al., 2016a). That is, useful content satisfies users’ needs. According to ARM, positive emotions typically arise as reactions to stimuli in an individual’s environment that he or she appraises as satisfying his or her needs, goals, or concerns (Zhang, 2013). Following this logic, we propose that that usefulness of content information as an environmental stimulus induces positive emotions and thereby promotes liking behavior on WeChat.

*Content interestingness* is the extent to which the content provided by a poster is enjoyable in its own right apart from content usefulness (Olney et al., 1991; Zhao et al., 2016a). On WeChat, users read others’ posts for pleasure and joy, and this enjoyable content can satisfy their entertainment needs. Previous research indicates that users’ positive emotional states are aroused when they experience enjoyment (Olney et al., 1991). Thus, the entertainment benefits obtained from content cause users to experience positive emotional changes, which in turn promote their liking behavior.

H4: Information usefulness influences WeChat users’ positive emotions.
H5: Information interestingness influences WeChat users’ positive emotions.

### Positive Emotions and Like Intention

Users’ liking behavior on social media is often aroused by emotion (Sun et al., 2016; Gan, 2017). That is, an important reason for clicking the like button on social media is to express aroused emotional responses (Sun et al., 2016). According to ARM, affective cues can influence people’s emotions and subsequently generate or arouse people’s behavior tendencies (Zhang, 2013). In other words, emotional response mediates the impact of affective cues on behavior tendencies. Thus, users’ decision making and behaviors will differ when their emotional states differ. In social media, people click Like button to show their appreciation to others or their posts, thus, positive emotion induce his/her Liking behavior. Positive emotions refer to “the extent to which a person feels enthusiastic, excited, and inspired” (Verhagen and Dolen, 2011). These emotional responses are widely considered key predictors of people’s behavior. For example, Wang et al. (2017) find that positive emotions positively affect users’ information-sharing behavior. Verhagen and Dolen (2011) show that positive emotions can improve customers’ purchase intentions. In line with these studies and ARM, we propose the following:

H6: Positive emotions positively influence WeChat users’ intention to like.

### Expected *Guanxi* Benefits From Positive Emotions and Like Intention

In addition to expressing positive emotions, users click on the like button to enhance and maintain *guanxi* with others (Wang et al., 2016). Thus, expected *guanxi* benefit is also a key predictor of liking behavior. Expected *guanxi* benefit reflects the degree to which an individual can maintain or improve interpersonal *guanxi* through liking behavior (Bock et al., 2005). People often use social media to enhance and improve their relationship with others, and social media platforms provide many functions to help them do so, including the ability to click the like button, write reviews, and send instant messages via text and voice (Wang et al., 2016). As noted previously, many users treat the number of likes received from others as a reflection of their reputation and social status (Sun et al., 2016); thus, people often use the like button to show their support of others or others’ posts, enabling them to maintain and enhance *guanxi* with others. We propose that users may engage in liking behavior when they expect to obtain a high *guanxi* benefit through such behavior.

Expected *guanxi* benefit also acts as an affective cue that may influence positive emotions. Previous research indicates that individuals will feel positive emotions about a behavior when they can benefit from that behavior. For example, Ajzen and Fishbein (1980) show that an individual’s positive emotions become aroused when he or she gains benefits from social behavior.

Thus, we propose the following:

H7: Expected *guanxi* benefit positively influences WeChat users’ liking intention.
H8: Expected *guanxi* benefit positively influences WeChat users’ positive emotions.

**FIGURE 1:**
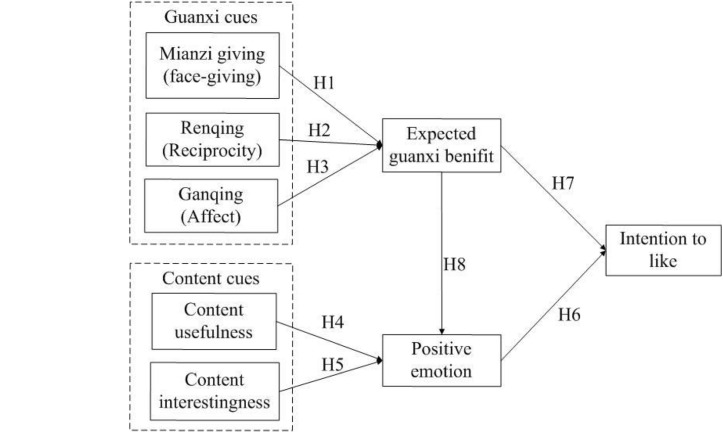
Conceptual model.

Figure 1 shows the conceptual model of this study.

## Methodology

### Measurement

Our research model consists of eight multi-item constructs, and 28 measurement items were generated (see Table 1). We developed all instruments by adapting existing validated instruments when necessary. In particular, we adapted the measurement scales for the three dimensions of *guanxi* (*mianzi* giving, *renqing*, and *ganqing*) from Lee and Dawes (2005) and Nie et al. (2011). Scales to measure content usefulness came from Sussman and Siegal (2003), and those for content interestingness came from Olney et al. (1991). Items for expected *guanxi* benefit came from Hsu and Lin (2008). We adapted items of positive emotion from Beatty and Ferrell (1998). Finally, we adapted items of intention to like from Verhoef et al. (2002). All items were measured on a 7-point Likert scale (1 = strongly disagree, 7 = strongly agree).

**TABLE 1:** Constructs and measures.

Constructs	Measures	Sources
*Mianzi* giving (M)	M1. This friend in WeChat care for Mianzi (face)	Lee and Dawes, 2005; Nie et al., 2011
	M2. The more “likes” we receive, the more Mianzi (face) we have.	
	M3. We give Mianzi (face) to our friend in WeChat, and he/she also gives us Mianzi.	
*Renqing* (R)	R1. In the WeChat, we will do this friend a favor by clicking like button if he/she did one for us before.	Lee and Dawes, 2005; Nie et al., 2011
	R2. In the WeChat, this friend will do us a favor by clicking like button if we did one for him/her before.	
	R3. In the WeChat, I feel a sense of obligation to this friend for doing him/her a favor by clicking like button.	
*Ganqing* (G)	G1. This friend is my closeness friend, and we care for each other wholeheartedly	Lee and Dawes, 2005; Nie et al., 2011
	G2. I like this friend, and he/she likes me.	
	G3. I would try my best to help this friend when he/she is in need because he/she is a closeness friend of mine.	
Usefulness (U)	U1: The information in this post is important.	Sussman and Siegal, 2003
	U2: The information in this post is informative.	
	U3: The information in this post is helpful.	
Interestingness (I)	I1: The information in this post is curious.	Olney et al., 1991
	I2: The information in this post is boring.	
	I3: The information in this post is interesting.	
	I4: The information in this spot is attractive.	
Expected *guanxi* benefit (EGB)	EBG1: Clicking like button on a friend’s post in WeChat will strengthen the guanxi between us.	Hsu and Lin, 2008
	EBG2: Clicking like button on a friend’s post in WeChat will create new guanxi with him/her.	
	EBG3: Clicking like button on a friend’s post in WeChat will increase the trust between us.	
Positive emotion (PE)	PE1. When clicking like button on that post in WeChat, I was excited.	Beatty and Ferrell, 1998
	PE2. When clicking like button on that post in WeChat, I was I was enthusiastic.	
	PE3. When clicking like button on that post in WeChat, I was inspired.	
Intention to like (IL)	IL1. I am likely to click like button on this post.	Verhoef et al., 2002
	IL2. I would love to click like button on this friend’s post continuously.	
	IL3. I predict that I shall click like button on this post.	

### Data Collection

The online survey took place in October 2017 on WeChat. The survey was available on the website for a 2-month period and ended when no new responses were generated (see Appendix A). We recruited 10 WeChat users to independently send invitation messages with a link to the online questionnaire to their friends on WeChat. At the same time, their friends also were asked to repost the survey on their social networks. Participants were directed to read a short description of study when they opened the link, then they were asked launch their WeChat and click into the Moments on Discover page. They were then asked to read the latest post in the Moments, go back to the survey and answer questions. To encourage participation, valid respondents were offered incentives of 10 RMB to complete the survey. In total, 365 respondents completed the questionnaires. We checked all responses and dropped 23 responses that had the same score for all items. We deleted another 15 responses because of missing data. As a result, 327 valid responses were processed for further analysis. Table 2 presents the demographic information of the respondents.

**TABLE 2:** Demographics of the research sample.

Variable		Count	%
Gender	Male	183	56.0
	Female	144	44.0
Age	17 or bellow	6	1.8
	18–30	154	47.1
	31–40	103	31.5
	41–50	60	18.3
	>51	4	1.2
Education level	Junior high school	10	3.1
	Senior high school	15	4.6
	Undergraduate	262	80.1
	Postgraduate	40	12.2
Monthly income	<2,000 Yuan	32	9.8
	2,000–3,999 Yuan	97	29.7
	4,000–5,999 Yuan	101	30.9
	60,000–7,999 Yuan	71	21.7
	>≥8,000 Yuan	26	8.0
Occupation	Student	65	19.9
	Company employee	93	28.4
	Employee in public institutions	84	25.7
	Civil servant	33	10.1
	Self-employed	38	11.6
	Retired	10	3.1
	Others	4	1.2
Length of use	<6 months	8	2.4
	>6 months and <1 year	35	10.7
	>1 year and <2 years	124	37.9
	>2 years and <3 years	89	27.2
	>3 years	71	21.7
Average minutes using WeChat per day	<10 min	86	26.3
	>10 min and <30 min	101	30.9
	>30 min and <60 min	104	31.8
	>60 min	36	11.0

## Data Analysis and Results

We employed partial least squares-structural equation modeling (PLS-SEM) using SmartPLS 2.0 M3 to estimate the parameters in the measurement and structural part of the model. PLS-SEM is a useful approach because of the minimal demands on measurement scales, sample size, and residual distributions. An additional benefit of PLS is its ability to screen out factors that have a negligible effect on the dependent variables. PLS-SEM is thus appropriate for this study.

### Analysis of Measurement Models

Table 3 shows the results of the measurement models in PLS-SEM analysis. All item loadings were greater than 0.7, with a significant *t*-value, and the average variance extracted (AVE) for every construct was greater than 0.5, indicating convergent validity of all constructs. All Cronbach’s alpha and composite reliability (CR) values exceed the general acceptable value 0.7, suggesting good reliability. Finally, as Table 4 shows, the square root of the AVE for each construct is greater than its correlations with other variables, demonstrating adequate discriminant validity of all the constructs.

**TABLE 3:** Scale properties.

Factor	Item	Standardized loading	Cronbach’s alpha	Composite reliability	AVE
*Mianzi* giving	M1	0.872	0.822	0.894	0.739
	M2	0.793			
	M3	0.909			
*Renqing*	R1	0.916	0.889	0.931	0.819
	R2	0.873			
	R3	0.924			
*Ganqing*	G1	0.928	0.934	0.958	0.884
	G2	0.937			
	G3	0.956			
Usefulness	U1	0.929	0.908	0.942	0.844
	U2	0.937			
	U3	0.890			
Interestingness	I1	0.890	0.917	0.941	0.801
	I2	0.885			
	I3	0.930			
	I4	0.874			
Expected *guanxi* benefit	EGB1	0.887	0.913	0.946	0.852
	EGB2	0.957			
	EGB3	0.924			
Positive emotion	PE1	0.806	0.780	0.872	0.695
	PE2	0.791			
	PE3	0.899			
Intention to like	IL1	0.976	0.983	0.989	0.967
	IL2	0.988			
	IL3	0.987			

**TABLE 4:** Correlation coefficient matrix and square roots of AVEs.

	M	R	G	U	I	EGB	PE	IL
M	0.86							
R	0.51	**0.90**						
G	0.56	0.66	**0.94**					
U	0.51	0.57	0.60	**0.92**				
I	0.58	0.56	0.56	0.61	**0.89**			
EGB	0.46	0.54	0.56	0.63	0.64	**0.92**		
PE	0.60	0.64	0.69	0.61	0.64	0.67	**0.83**	
IL	0.47	0.58	0.58	0.46	0.53	0.47	0.59	**0.98**

*Bolded values are the square roots of AVEs.*

### Structural Model Test

**FIGURE 2:**
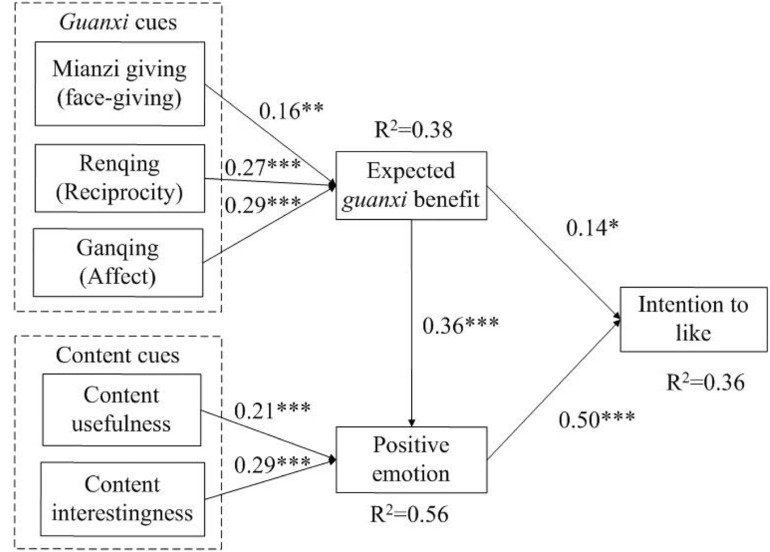
Model testing results by SmartPLS. *p < 0.05, **p < 0.01, ***p < 0.001; n.s, not significant.

Figure 2 shows the results of model testing, including path coefficients, the corresponding levels of significance, and variance explained. An adequate proportion (36%) of the variance in intention to like is explained by the proposed model. As expected, positive emotions (β = 0.50, *t* = 8.15, *p* < 0.001) and expected *guanxi* benefit (β = 0.14, *t* = 2.18, *p* < 0.05) positively affect intention to like. Thus, H6 and H7 were supported. For the antecedents of positive emotions, expected *guanxi* benefit (β = 0.36, *t* = 5.61, *p* < 0.001), content usefulness (β = 0.21, *t* = 4.25, *p* < 0.001), and content interestingness (β = 0.29, *t* = 5.77, *p* < 0.001) have significant effects on positive emotions; thus, H8, H4, and H5 were supported. Overall, a significant proportion (56%) of the variance in positive emotions is explained by all affective cues (i.e., expected *guanxi* benefit, content usefulness, and content interestingness). In addition, *mianzi* giving (β = 0.16, *t* = 2.98, *p* < 0.05), *renqing* (β = 0.27, *t* = 4.56, *p* < 0.001), and *ganqing* (β = 0.29, *t* = 4.53, *p* < 0.001) exert significant effects on expected *guanxi* benefit, in support of H1, H2, and H3, respectively. The explained variance of expected guanxi benefit is 38%.

## Discussion

This study tests a conceptual model that proposes that content cues (i.e., content usefulness and content interestingness) affect WeChat users’ positive emotions, which in turn predict their intention to like. More important, this study proposes that *guanxi* cues (i.e., *mianzi* giving, *renqing*, and *ganqing*) positively affect WeChat users’ expected *guanxi* benefit, which in turn affects intention to like in two ways. On the one hand, expected *guanxi* benefit positively influences intention to like directly; on the other hand, expected *guanxi* benefit translates into positive emotions, which in turn predict users’ intention to like. The results provide support for all the research hypotheses. Specifically, positive emotions and expected *guanxi* benefit are two crucial facilitators of intention to like on WeChat. The findings corroborate previous assumptions that users click the like button not only to express their positive emotions about the content but also to maintain or enhance relationships with others. In terms of the antecedents, expected *guanxi* benefit, content usefulness, and content interestingness exert positive effects on positive emotions. In addition, *mianzi*, *renqing*, and *guanxi* have positive effects on expected *guanxi* benefit.

### Theoretical Implications

This study contributes to extant literature in two ways. First, the Chinese use WeChat both to express their emotions when reading useful and interesting content and to strengthen *guanxi* with their friends. By contrast, prior research neglects the effect of *guanxi* on users’ liking behavior (Mariani and Mohammed, 2014; Basalingappa et al., 2016; Sun et al., 2016; Gan, 2017). We show that *guanxi* cues (i.e., *mianzi* giving, *renqing*, and *ganqing*) motivate Chinese users to click the like button when they want to obtain a high level of the *guanxi* benefit through liking behavior. Thus, this study contributes to the literature in this field by treating *guanxi* as a key antecedent of liking behavior in the Chinese context.

Second, previous research mainly focuses on the factors that affect users’ likelihood to click the like button, while overlooking the underlying mechanisms that may affect users’ intention to click. This study aimed to fill these gaps by integrating *guanxi* theory and ARM to assess users’ liking behavior. We proposed that positive emotions and expected *guanxi* benefit would arouse users’ liking behavior. More specifically, we expected useful and interesting content to arouse users’ positive emotions, which in turn motivate their intention to like. In addition, *guanxi* cues (i.e., *mianzi* giving, *renqing*, and *ganqing*) motivate Chinese users’ liking behavior in two ways. On the one hand, they positively affect expected *guanxi* benefit and, in turn, influence intention to like directly. On the other hand, *guanxi* cues transfer to expected *guanxi* benefit and thereby produce positive emotions, in turn influencing intention to like. We enrich the literature on liking behavior by showing that positive emotions and expected *guanxi* benefit are the two underlying mechanisms of Chinese consumer liking behavior. In addition, we add to the literature by applying *guanxi* theory and ARM in a new context.

Third, previous research has focused on social benefits, social support and their impact on WeChat users’ liking behavior (Sun et al., 2016; Gan, 2017). However, little research has approached liking behaviors on WeChat in a affective or social image perspective. The current research introduced the concept of *guanxi*, and examined *mianzi* (face) giving and *ganqing* (affect) on liking behavior in WeChat. The results indicated that people like each other’s post on WeChat so as to create or maintain a desirable social image (*mianzi* face giving) and express their affect for close friends (*ganqing*). Thus, this study contributes to the field of liking behavior on social media by introducing the concept of *guanxi* and demonstrated the impact of social image concern and affective concerns on liking behavior.

### Practical Implications

From a practical standpoint, the findings of this study are also important for social media developers to encourage Chinese users to adopt liking behavior. First, Chinese users’ liking behavior can be motivated by positive emotions; that is, Chinese people are more likely to click the like button when they experience enjoyment and are in a positive emotional state. Thus, social media developers can improve Chinese users’ liking behavior by designing various features to cultivate their positive emotions, such as designing the content recommendation function to provide useful and interesting content ranking lists to users, which will arouse their positive emotions. Second, Chinese users’ liking behavior is also driven by motivations to maintain and enhance *guanxi*, with *mianzi* giving, *renqing*, and *ganqing* improving the expected *guanxi* benefit. Thus, to improve users’ *mianzi* giving motivation, social media developers can design features to induce users’ *mianzi* consciousness on social media (e.g., informing their friends when their posts are liked by others) and provide like ranking lists to all users. In addition, social media developers can cultivate *renqing* and *ganqing* by offering more channels to improve interpersonal interaction.

### Limitation and Future Research Direction

This study also has two limitations. First, we conducted the study in China; however, the motivations underlying people’s use of the like button on social media may differ across cultures. Although *guanxi* is a uniquely Chinese cultural construct, we assume that its underlying components such as reciprocity (*renqing*), affect (*ganqing*), social image support (*mianzi* face giving) should also exist and work in other culture. It would be interesting to see future research examining these underlying components and their impacts on social media liking behavior in Western culture. Second, although users’ behavior is influenced by *guanxi* and content, liking behavior is also potentially influenced by people’s personalities. Future research should consider how personal characteristics influence liking behavior.

## Data Availability Statement

The datasets generated for this study are available on request to the corresponding author.

## Ethics Statement

Ethical review and approval was not required for the study on human participants in accordance with the local legislation and institutional requirements. Written informed consent for participation was not required for this study in accordance with the national legislation and the institutional requirements.

## Author Contributions

HZ and MZ conceived of the presented idea, developed the theory and research model, collected data, and performed the analysis. Both authors contributed to the article and approved the submitted version.

## Conflict of Interest

The authors declare that the research was conducted in the absence of any commercial or financial relationships that could be construed as a potential conflict of interest.
