# Supplementary material for: The Role of Guanxi and Positive Emotions in Predicting Users’ Likelihood to Click the Like Button on WeChat
Source: Front Psychol. 2020 Jul 22;11:1736. doi: 10.3389/fpsyg.2020.01736 (PMC7387579; doi:10.3389/fpsyg.2020.01736)
Supplement: Supplementary file 1 [file Data_Sheet_1.doc]

**Appendix A**

**First please launch your WeChat and click into the Moments on Discover page. Then read the latest post in the Moments and answer the following questions.**

**Ⅰ. Please rate the latest post in the Moments according to the following statements. (1 = strongly disagree, 7 = strongly agree)**

1. I am likely to click like button on this post.
2. I would love to click like button on this friend’s post continuously.
3. I predict that I shall click like button on this post.
4. The information in this post is important.
5. The information in this post is informative.
6. The information in this post is helpful.
7. The information in this post is curious.
8. The information in this post is boring.
9. The information in this post is interesting.
10. The information in this post is attractive.

**Ⅱ. Please rate the following statements. (1 = strongly disagree, 7 = strongly agree)**

1. Clicking like button on a friend’s post in WeChat will strengthen the guanxi between us.
2. Clicking like button on a friend’s post in WeChat will creat new guanxi with him/her.
3. Clicking like button on a friend’s post in WeChat will increase the trust between us.
4. When clicking like button on this post in WeChat, I was excited.
5. When clicking like button on this post in WeChat, I was I was enthusiastic.
6. When clicking like button on this post in WeChat, I was inspired.

**Ⅲ. Please rate the following statements in terms of the relationship between you and the post author.**

1. This friend in WeChat cares about Mianzi(face).
2. The more likes we receive, the more Mianzi(face) we have.
3. We give Mianzi(face) to our friend in WeChat, and he/she also gives us Mianzi.
4. In WeChat, we will do this friend a favor by clicking like button if he/she did one for us before.
5. In WeChat, this friend will do us a favor by clicking like button if we did one for him/her before.
6. In WeChat, I feel a sense of obligation to this friend for doing him/her a favor by clicking like button.
7. This friend is my closeness friend, and we care for each other wholeheartedly.
8. I like this friend, and he/she likes me.
9. I would try my best to help this friend when he/she is in need because he/she is a closeness friend of mine.

**Ⅳ. Basic information**

1. How long have you been using Moments in WeChat?

Less than 6 months

Over 6 months and less than one year

Over one year and less than two years

Over two years and less than three years

Over three years

1. How much time do you spend on WeChat everyday?

Less than 10 minutes

Over 10 minutes and less than 30 minutes

Over 30 minutes and less than 60 minutes

Over 60 minutes

1. Your gender:

Male

Female

1. Your age:

Below 18

18-30

31-40

41-50

Over 50

1. Your occupation:

Student

Employee of enterprises

Employee of public institutions or civil servant

Farmer

Freelancer

Unemployed or retired

Others

1. Your education level:

Junior high school or below

Senior high school

Undergraduate

Postgraduate or above

1. Your monthly income:

Below 2000 Yuan

2000-3999 Yuan

4000-5999 Yuan

6000-7999 Yuan

8000 Yuan or above
